# Crocetin Alleviates Inflammation in MPTP-Induced Parkinson's Disease Models through Improving Mitochondrial Functions

**DOI:** 10.1155/2020/9864370

**Published:** 2020-10-10

**Authors:** Na Dong, Zhong Dong, Ying Chen, Xiaosu Gu

**Affiliations:** ^1^Department of Neurology, Tianjin Huanhu Hospital, Tianjin 300350, China; ^2^Department of Neurology, Affliated Hospital of Nantong University, Nantong, Jiangsu 226001, China

## Abstract

Parkinson's disease (PD) is the second most common neurodegenerative disease. Crocetin, derived from saffron, exerts multiple pharmacological properties, such as anti-inflammatory, antioxidant, antifatigue, and anticancer effects. However, the effect of crocetin on PD remains unclear. In this study, we designed experiments to investigate the effect of crocetin against MPTP-induced PD models and the underlying mechanisms. Our results showed that crocetin treatment attenuates MPTP-induced motor deficits and protects dopaminergic neurons. Both *in vivo* and *in vitro* experiments demonstrated that crocetin treatment decreased the expression of inflammatory associated genes and inflammatory cytokines. Furthermore, crocetin treatment protected mitochondrial functions against MPP+ induced damage by regulating the mPTP (*mitochondrial permeability transition pore*) viability in the interaction of ANT (*adenine nucleotide translocase*) and Cyp D (*Cyclophilin D*) dependent manner. Therefore, our results demonstrate that crocetin has therapeutic potential in Parkinson's disease.

## 1. Introduction

Parkinson's disease (PD) is the second most common neurodegenerative disease after Alzheimer's disease and affects more than 4 million population [1]. The clinical symptoms of PD include resting tremor, bradykinesia, muscle stiffness, and postural instability [2]. Some chemical compounds like ursolic acid and chlorogenic acid exhibit potent anti-Parkinsonian activity in MPTP-induced Parkinsonian mouse model [3–5]. It is disappointing that the currently available medications are still restricted to the symptomatic relief of PD.

**Figure 1:**
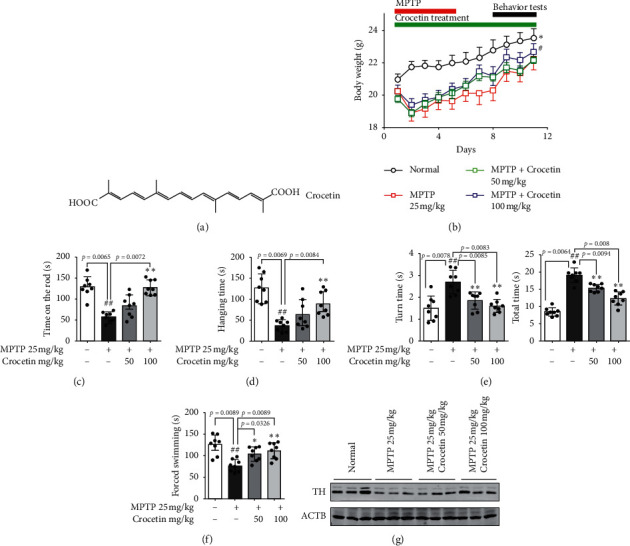
Crocetin ameliorates MPTP-induced motor activity impairments. C57BL/6 mice treated with MPTP (25 mg/kg/d, 5 days, i.p.) were orally administered with crocetin for 11 days. (a) The structure of crocetin. (b) Weight change was monitored throughout the experiment. Outcomes of the rotarod performance test (c) and hanging performance (d) on day 8 and day 9. Bar graphs reveal the latency to fall in seconds (s). (e) Time to turn and total time in pole test was recorded on day 5 after the cease of MPTP treatment. (f) The total time of forced swimming was recorded. (g) Western blot determination of tyrosine hydroxylase (TH) expression levels in the striatum of each group. Data are expressed as the mean ± standard deviation, and statistical analysis was performed using a one-way analysis of variance followed by Tukey's post hoc test. ^#^*P* < 0.05, ^##^*P* < 0.01 versus normal; ^*∗*^*P* < 0.05, ^*∗∗*^*P* < 0.01 versus MPTP, *n* = 8 mice in each group.

Crocetin is a natural apocarotenoid dicarboxylic acid derived from saffron [6, 7]. The structure of crocetin contains a 20-carbon chain, six double bonds, and two carboxylic acid groups (Figure 1(a)). A large number of reports have been demonstrated that crocetin exerts multiple pharmacological properties, such as anti-inflammatory, antioxidant, antifatigue, and anticancer effects [8–11]. In addition, crocetin was demonstrated to play neuroprotective effects on cerebral ischemia [12]. Tiribuzi et al. found that transcrocetin could improve amyloid-*β* degradation in monocytes from Alzheimer's disease patients [13]. Zhang et al. reported that crocetin was able to significantly reduce amyloid-*β* (A*β*) 40 and A*β*42 secretion in Hela cells without affecting cell viability. Moreover, crocetin attenuated the proinflammatory cytokines and enhanced anti-inflammatory cytokine in plasma in APPsw transgenic mice [7]. The previous studies indicated that crocetin played key roles in various diseases, including neurodegenerative diseases. However, whether crocetin has an effect on PD remains unclear.

In the current study, we designed experiments to investigate the effect of crocetin MPTP-induced PD models and the underlying mechanisms. Our results showed that crocetin could attenuate MPTP-induced motor deficits and protect dopaminergic neurons. Moreover, crocetin was able to alleviate inflammation in MPTP-induced PD mice and BV2 cells. More importantly, crocetin alleviated inflammation and improved MPTP-induced motor deficits via the improvement of mitochondrial functions.

## 2. Materials and Methods

### 2.1. Reagents

Crocetin and MPTP were purchased from Sigma (St. Louis, MO). AM1241 was obtained from Selleck (Shanghai, China). Dimethyl sulfoxide (DMSO) and Triton X-100 were obtained from Fisher Scientific (Pittsburgh, PA). All the antibodies were from Abcam (Cambridge, MA) or Cell Signaling Technology (Danvers, MA). All other chemicals, unless otherwise stated, were from Sigma (St. Louis, MO).

### 2.2. Animals and MPTP Treatment

All experiments were carried out in male C57BL/6 mice, weighing 25–30 g at the time of exposure. Animals were housed at 22 ± 1°C (12-hour light-dark cycle) with ad libitum access to food and water for one week before experiments. All experiments were carried out following the National Institutes of Health Guide for the Care and Use of Laboratory Animals and approved by the Institutional Animal Care and Use Committee of Affiliated Hospital of Nantong University. Animals were divided into four experimental groups: the normal group, MPTP-treated group, MPTP + crocetin (50 mg/kg) group, and MPTP + crocetin (100 mg/kg) group (8 mice per group). Mice received intraperitoneal (i.p.) injections of MPTP (30 mg/kg, i.p.) and oral administration of crocetin once a day following previously published guidelines [14].

### 2.3. Behavioral Tests

We performed a rotarod, suspension test, pole test, and forced swimming 3 days after the last MPTP injection. The rotarod test is widely used to generally assess motor performance, which was done as described previously and modified a little [15]. Mice were trained for two consecutive days before MPTP injections in acceleration mode (2–20 rpm) over 5 min. The training was repeated at a fixed speed (15 rpm) until the mice were able to stay on the rod for at least 150 s. After MPTP and crocetin treatment, the time falling down from rod was recorded in acceleration mode (2–20 rpm). For suspension experiment, C57BL/6J mice were placed on a horizontal wire of ∼1.5 mm in diameter, suspended 30 cm from the ground, and the hang time was recorded to detect mouse limb coordination. The pole test is used to assess motor function and was done as described previously [16]. Mice were placed facing upwards at the top of a pole (1 cm in diameter, 45 cm in length) that had been covered with surgical tape to provide a rough surface and capped with a flat plastic disc to prevent the mouse from traversing the top of the pole. The times for the mouse to turn and face downwards (turn time) and for the mouse to descend into the home cage (total time) were recorded. Mice were trained on the task for two days before MPTP treatment. The test trial was performed three times per animal, and average values from three examinations were used for each animal. For the forced-swimming test, C57BL/6J mice in each group were dropped individually into glass cylinders (10 × 10 cm) containing 10 cm deep water that maintained at 25 ± 1°C and remained for 6 min. The time of swimming was recorded during the last 4 min of the 6-min testing period, followed by 2 min of habituation.

### 2.4. Western Blot

The brain tissues of mice and cells were homogenized in lysis buffer containing protease inhibitors. The homogenate was centrifuged at 14000 g for 15 min at 4°C and the protein concentration was determined using the BCA kit. 30 *μ*g lysate was loaded onto 10% SDS-PAGE. The proteins were transferred to PVDF membranes (Millipore, MA, USA). The membranes were blocked for 1 h in 5% dry milk and then incubated overnight with one of the following primary antibodies: anti-iNOS (ab178945), anti-Pro-caspase-1 (ab179515), anti-ANT (ab102032), anti-Cyp D (ab16045) (Abcam, Cambridge, MA, USA), anti-TH (#2792), anti-COX2 (#12282), anti-p-p65 (#3033), anti-Cleaved-caspase-1 (#89332), anti-Cyto C (#4280), anti-VDAC (#4866), anti-COX4 (#4850), or anti-*β*-actin (#3700) (Cell Signaling Technology, Beverly, USA). After washing 3 times in TBST for 5 min each, the membranes were incubated with goat anti-mouse, anti-rabbit, or anti-rat HRP for 1 h at room temperature. Then, the membranes were washed 3 times in TBST for 5 min each. The signal was visualized using an ECL chemiluminescence kit (Amersham Biosciences/GE Healthcare; Piscataway, NJ).

### 2.5. Cell Culture

BV2 cells were purchased from ATCC and incubated in Dulbecco's modified Eagle's medium (DMEM) with 10% fetal bovine serum (FBS, Invitrogen) at 37°C in 5% CO_2_.

### 2.6. Cell Viability

BV2 cells were treated with 0.5 mM MPP+ for 6 h, then 2.5 *μ*M, 5 *μ*M, and 10 *μ*M crocetin were added and incubated for another 24 h. After treatment, the cells were mixed with 10 *μ*L of Cell Counting Kit-8 (CCK-8) solution per well. After incubation for further 2 h at 37°C, the optical density was measured for absorbance at 450 nm by a microplate reader.

### 2.7. Immunofluorescence

The method of immunofluorescence in this study was used as described previously [17]. Briefly, tissue samples were fixed in 4% paraformaldehyde (PFA). The tissue samples were embedded in paraffin and cut into sections. Then, the sections were incubated with the following primary antibody: anti-Iba-1 at 4°C overnight. In addition, donkey anti-rabbit-Alexa Fluor 488 was used to stain the sections for 1 h at 25°C. Finally, we used an Olympus Fluoview FV1000 to shoot the immunofluorescence slides.

### 2.8. Real-Time Quantitative PCR (RT-qPCR) Analysis

Total RNA was isolated from striatum tissue or BV2 cells using Trizol reagent (Invitrogen) according to the manufacturer's protocols. cDNA was synthesized with the PrimerScript Reverse Transcriptase Kit (Takara). RT-qPCR was performed using SYBR Premix Ex Taq™ (Takara) on the QuantStudio 7 Flex Real-Time PCR System. The primer sequences (Sangon Biotech, China) are listed as Table 1 [18].

### 2.9. ELISA

The protein level of TNF-*α* and IL-1*β* in PD mice treated with or without crocetin was detected by ELISA (Beyotime Biotechnology) according to the manufacturer's protocols.

### 2.10. Determination of Intracellular ROS

The 2′, 7′-dichlorofluorescein diacetate (DCFH-DA) was used to monitor intracellular ROS levels. BV2 cells were treated with 2.5 *μ*M, 5 *μ*M, and 10 *μ*M crocetin for 12 h, then MPP+ (0.5 mM) was added for another 24 h. Then, the cells were then treated with DCFH-DA (5 *μ*M) and the fluorescence intensity of the treated cells was measured by flow cytometry.

### 2.11. Mitochondrial Membrane Potential (MMP)

BV2 cells were pretreated with 2.5 *μ*M, 5 *μ*M, and 10 *μ*M crocetin, then MPP+ (0.5 mM) was added. The cells were incubated with JC-1 (1 *μ*g/mL) in culture medium at 37°C for 30 min and then were imaged by fluorescence microscopy (Olympus Fluoview FV1000). The ratio of red/green fluorescence intensity was analyzed by the microplate reader.

### 2.12. ATP Measurement

5 × 10^5^ BV2 cells seeded into 6-cm dishes were treated with 0.1 mM, 0.3 mM, and 1 mM crocetin for 12 h, then MPP+ (2.5 mM) was added, and cells were incubated for another 24 h. The cell lysates were immediately prepared by the ATP detection kit (S0027, Beyotime, China) according to the manufacturer's instructions. Chemiluminescence for ATP content was read in an FL *×* 800 microplate fluorescence reader (Biotek, Winooski, VT, USA).

### 2.13. Calcium Detect

BV2 cells were pretreated with 2.5 *μ*M, 5 *μ*M, and 10 *μ*M crocetin, before MPP+ (0.5 mM). Then, the cells were washed and simultaneously incubated for 1 h with the fluo-4-acetoxymethyl ester (Fluo-4AM) to monitor cytosolic free calcium. The fluorescence of Fluo-4AM was measured by a fluorescence microplate reader.

### 2.14. Mitochondria Purification

The isolation of mitochondria from the brain of mice was done as described previously [20]. Brains from C57BL/6 mice were homogenized in isolation buffer (225 mM mannitol, 75 mM sucrose, 1 mM EGTA, 5 mM HEPES, and 2 mg/ml fat-free BSA) using a motorized Dounce homogenizer with eight up-and-down strokes. The homogenate was centrifuged at 1,000 g for 10 minutes, and the resulting supernatant was layered onto 5 ml of 7.5% Ficoll medium on top of 5 ml of 10% Ficoll medium and centrifuged at 79,000 g for 30 minutes (the Ficoll medium contained 0.3 M sucrose, 50 *μ*M EGTA, and 10 mM HEPES). The mitochondrial pellet was resuspended in an isolation buffer. Protein concentrations were determined by the BCA method.

### 2.15. Mitochondrial Swelling

Isolated mitochondria were treated with buffer (70 mM sucrose, 230 mM mannitol, 3 mM HEPES, 2 mM Tris-phosphate, 5 mM succinate, and 1 *μ*M rotenone). Then, mitochondrial swelling was measured in absorbance at 540 nm.

### 2.16. Statistical Analysis

All statistical analysis was performed with GraphPad Prism 7 software (Version 7.00; GraphPad Software, Inc., San Diego, CA). One-way analysis of variance (ANOVA) followed by Tukey's multiple comparisons test was used for experiments with multiple groups. All data are presented as mean ± SD, and the criterion of significance was set at *P* < 0.05.

## 3. Results

### 3.1. Crocetin Attenuates MPTP-Induced Motor Deficits and Protects Dopaminergic Neurons

After the injection of MPTP, the loss of weight was monitored every day. As shown in Figure 1(b), MPTP-induced PD model mice had a loss of weight compared to the control group, which was reversed following treatment with crocetin in PD mice. Motor deficits and bradykinesia occur in the majority of patients with PD and MPTP-induced mice models of PD. Therefore, we implemented a series of behavioral tests such as rotarod, suspension, and pole test on day 3 after the final MPTP injection. An overall difference between normal and MPTP-treated PD mice was found in the rotarod performance. Mice in the crocetin-treated PD group had an obvious longer dropping latency than those of the MPTP group, which confirms that crocetin improved motor coordination deficits (Figure 1(c)). As shown in Figure 1(d), MPTP administration resulted in a decrease in neuromuscular strength as evidenced by the reduction in hanging time as compared to control. Pretreatment with crocetin to MPTP-treated mice distinctly enhanced hanging time as compared to the MPTP group. In the pole test, the MPTP group took significantly longer to turn down and to climb the pole than the control group. PD mice suffered from crocetin reduced turning time and climbing times (Figure 1(e)). It also has been mentioned that a depressive phenotype was observed in the PD mouse model [20]. Therefore, a forced-swimming test was used to detect depressive behavior. As shown in Figure 1(f), MPTP-induced PD mice significantly decreased swimming time than normal mice, and crocetin treatment could increase swimming time. Because TH expression is significantly attenuated in the brains of MPTP-induced PD model [21], we evaluated TH levels to confirm the establishment of a chronic MPTP-induced Parkinsonism mouse model and the protective effects of crocetin. In the striatal regions, TH levels were noticeably reduced in the MPTP group relative to the normal group. Crocetin attenuated the decrease in TH, suggesting that crocetin protected dopaminergic neurons against MPTP damage (Figure 1(g)). In a word, crocetin could attenuate MPTP-induced motor deficits and protect dopaminergic neurons *in vivo*.

### 3.2. Crocetin Alleviates Inflammation in MPTP-Induced PD Mice

**Figure 2:**
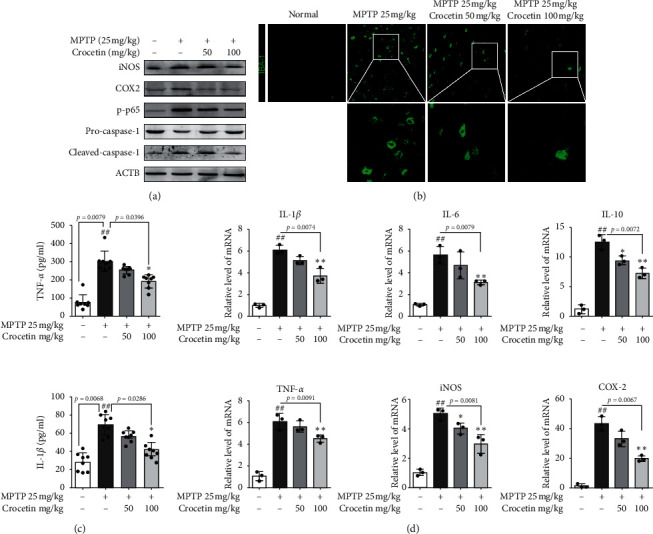
Crocetin inhibits inflammation in the MPTP-induced PD model. (a) The protein level of iNOS, COX2, p-p65, pro-caspase-1, and cleaved-caspase-1 in the striatum of crocetin-treated PD mice was measured using Western blot assay. (b) The expression of Iba-1 in the striatum was detected using the immunofluorescent assay. (c) The level of TNF-*α* and IL-1*β* in the serum of crocetin-treated PD mice was detected by ELISA. (d) The mRNA level of IL-1*β*, IL-6, IL-10, TNF-*α*, iNOS, and COX-2 in the striatum were measured using RT-qPCR. Data are expressed as the mean ± standard deviation, and statistical analysis was performed using a one-way analysis of variance followed by Tukey's post hoc test. ^##^*P* < 0.01 versus control; ^*∗*^*P* < 0.05, ^*∗∗*^*P* < 0.01 versus MPTP.

Because aberrant inflammatory response can result in nerve injury in activated microglia, we analyzed the expression of inflammatory associated genes and inflammatory cytokines [20]. The results of Western blotting showed that the levels of iNOS, COX2, p-p65, and Cleaved-caspase-1 were increased in MPTP-induced PD mice, while pretreatment with crocetin to MPTP-treated mice distinctly decreased the expression of iNOS, COX2, p-p65, and Cleaved-caspase-1 (Figure 2(a)). The Iba-1, a microglia marker, and fluorescent staining showed that the number of microglia was increased in MPTP-induced PD mice, and crocetin treatment could downregulate the number of microglia (Figure 2(b)). Besides, the protein levels of TNF-*α* and IL-1*β* were upregulated in MPTP-induced PD mice. Crocetin treatment reduced the protein levels of TNF-*α* and IL-1*β* in MPTP-induced PD mice (Figure 2(c)). Moreover, the RT-qPCR results showed that the relative mRNA expression levels of IL-1*β*, IL-6, IL-10, TNF-*α*, iNOS, and COX-2 were increased in MPTP-induced PD mice, whereas pretreatment with crocetin to MPTP-treated mice partially suppressed the expression of proinflammatory cytokines (Figure 2(d)).

### 3.3. Crocetin Inhibits MPP+ Induced Inflammation in BV2 Cells

**Figure 3:**
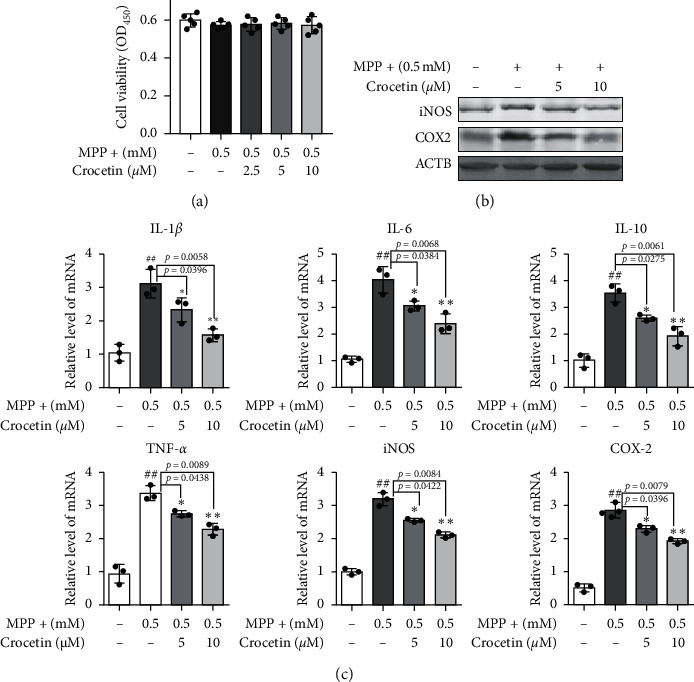
Crocetin inhibits MPP+ induced inflammation in BV2 cells. BV2 cells were treated with 0.5 mM MPP+ for 6 h, and then 2.5 *μ*M, 5 *μ*M, and 10 *μ*M crocetin were added and incubated for another 24 h. (a) Cell viability was determined using the CCK-8 assay. (b) The protein level of iNOS and COX-2 were measured using Western blot. (c) After different stimulation, the mRNA level of IL-1*β*, IL-6, IL-10, TNF-*α*, iNOS, and COX-2 in the BV2 cells was measured using RT-qPCR. Data are expressed as the mean ± standard deviation, and statistical analysis was performed using a one-way analysis of variance followed by Tukey's post hoc test. ^##^*P* < 0.01 versus control; ^*∗*^*P* < 0.05, ^*∗∗*^*P* < 0.01 versus MPP+.

As shown in Figure 3(a), crocetin at different concentrations (0, 2.5, 5, and 10 *μ*m) treatment did not show any cytotoxicity based on cell viability. The results of Western blotting showed that the levels of iNOS and COX-2 were increased in BV2 cells after stimulation with MPP+, and the effects were attenuated by crocetin (Figure 3(b)). Moreover, the mRNA expression levels of IL-1*β*, IL-6, IL-10, TNF-*α*, iNOS, and COX-2 were increased in BV2 cells after stimulation with MPP+, while crocetin treatment inhibited the changes (Figure 3(c)).

### 3.4. Crocetin Protects Mitochondrial Functions against MPP+ Induced Damage

**Figure 4:**
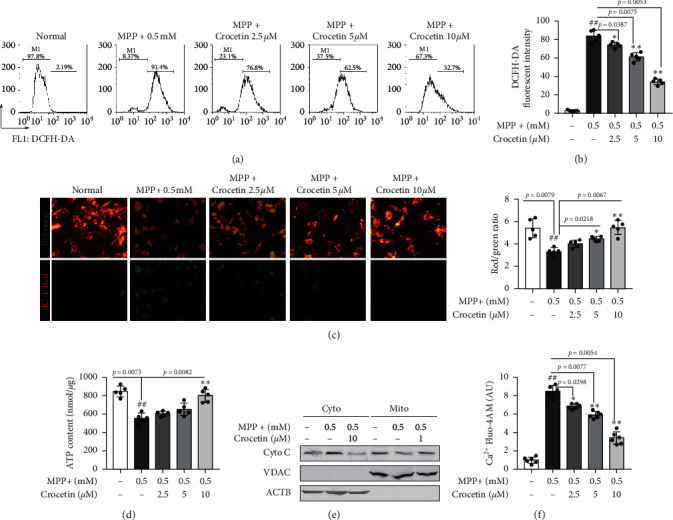
Crocetin improves mitochondrial dysfunctions in MPP + -damaged BV2 cells. BV2 cells were treated with 0.5 mM MPP + for 6 h, and then 2.5 *μ*M, 5 *μ*M, and 10 *μ*M crocetin were added and incubated for another 24 h. ((a), (b)) ROS was detected using DCFH-DA and measured by flow cytometer. (c) Mitochondrial membrane potential was measured using JC-1 dye and measured by a microplate reader. (d) Total ATP amounts in each group were measured. (e) The release of cytochrome c was measured between cytosol and isolated mitochondria. (f) The content of calcium in the cytosol was detected by Fluo-4AM and measured by a fluorescence microplate reader. Data are expressed as the mean ± standard deviation, and statistical analysis was performed using a one-way analysis of variance followed by Tukey's post hoc test. ^##^*P* < 0.01 versus control; ^*∗*^*P* < 0.05, ^*∗∗*^*P* < 0.01 versus MPP+. *n* = 5 in each group.

As shown in Figures 4(a) and 4(b), the intracellular ROS levels measured by DCFH-DA fluorescent intensity increased in BV2 cells after stimulation with MPP+, while crocetin treatment blocked the increase. The ratio of red/green fluorescence intensity of JC-1 dye indicated that MPP+ treatment significantly reduced the mitochondrial membrane potential (MMP), whereas the effect of MPP+ on MMP was reversed by crocetin (Figure 4(c)). In addition, MPP+ stimulation reduced the ATP content of BV2 cells, and the levels of ATP in BV2 cells were upregulated by crocetin after stimulation with MPP+ (Figure 4(d)). Moreover, the results of Western blotting showed that the levels of Cyto C in the cytoplasm were obviously increased by MPP+ stimulation, and the effects were attenuated by crocetin (Figure 4(e)). Furthermore, the content of calcium measured by Fluo-4AM was an increase in the cytosol of BV2 cells after stimulation with MPP+, while crocetin reduced the content of calcium (Figure 4(f)). These results suggest that crocetin has a potential protective effect in mitochondrial functions against MPP+ induced damage.

### 3.5. Crocetin Directly Inhibited the Open of mPTP through Blocking the Interaction of ANT and Cyp D

**Figure 5:**
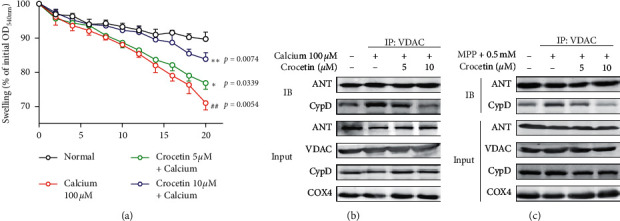
Crocetin blocks interaction between ANT and Cyp D to inhibit mPTP opening. (a) The curve is a typical sample of mitochondrial swelling induced by 100 *μ*M CaCl_2_. Mitochondrial swelling was measured by monitoring mitochondrial size at an absorbance of 540 nm. 5 *μ*M and 10 *μ*M final concentrations of crocetin were added to the mitochondrial suspension for 5 min before adding CaCl_2_. A change of absorbance within 20 min with a spectrophotometer indicated mitochondrial swelling. (b) Isolated mitochondria were pretreated with crocetin for 5 min followed by 100 *μ*M CaCl_2_ for 20 min, and then mitochondria were lysed and anti-VDAC antibody was added to pull down ANT and Cyp D. The levels of ANT and Cyp D pulled down by anti-VDAC were measured using immunoblotting. (c) BV2 cells were treated with 0.5 mM MPP+ for 6 h, and then 2.5 *μ*M, 5 *μ*M, and 10 *μ*M crocetin were added and incubated for another 24 h. The ANT and Cyp D pulled down by VDAC were detected using immunoblotting. Data are expressed as mean ± standard deviation, and statistical analysis was performed using a one-way analysis of variance followed by Tukey's post hoc test. ^##^*P* < 0.01 versus control; ^*∗*^*P* < 0.05, ^*∗∗*^*P* < 0.01 versus calcium group.

To investigate the effect of crocetin on mPTP viability, mitochondria isolated from mice brains were subjected to be treated with 100 *μ*M CaCl_2_ to trigger the opening of mPTP. CaCl_2_ treatment induced the mitochondrial swelling caused by the opening of mPTP, and the effect was blocked by crocetin (Figure 5(a)). To better understand the mechanism in which crocetin inhibited the opening of mPTP, we used a specific anti-VDAC mAb to coimmunoprecipitate with ANT and Cyp D. Crocetin treatment blocked the interaction of ANT and Cyp D in mitochondria treated with CaCl_2_ (Figure 5(b)). Moreover, Crocetin treatment also inhibited the interaction of ANT and Cyp D in BV2 cells treated with MPP+ (Figure 5(c)).

## 4. Discussion

Numerous natural products, like ursolic acid and chlorogenic acid, ameliorate neurobehavior in the MPTP-induced Parkinsonian mouse model [4, 5]. Moreover, ursolic acid also exhibits potent anti-inflammatory activity [3]. Similar to Ursolic acid and chlorogenic acid, preliminary data show that crocetin, natural organic acids, played the neuroprotective effects of neurodegenerative diseases [12, 13]. However, the role of crocetin in PD remains unclear. Herein, we demonstrated that crocetin treatment reversed a loss of weight of MPTP-induced PD model mice. Moreover, the effective effect of crocetin on motor deficits and bradykinesia, which occurs in the majority of patients with PD, was detected by rotarod, suspension, and pole test. The results indicated that crocetin significantly improved the motor deficits and bradykinesia of MPTP-induced PD model mice. Besides, crocetin treatment could increase swimming time, which has decreased in MPTP-induced PD mice. Given that the TH expression in the brains is an important marker of the MPTP-induced PD model [21], the TH levels were used to evaluate the protective effects of crocetin on MPTP-induced Parkinsonism mouse model. Crocetin attenuated the decrease in TH in the striatal regions, suggesting that crocetin protected dopaminergic neurons against MPTP damage. In a word, crocetin has neuroprotective effects on PD.

Neuroinflammation is one of the main features of PD and an undeniable phenomenon in the pathophysiology of PD [22]. Delattre et al. reported that maternal Omega-3 supplements can improve the dopaminergic system in pre- and postnatal inflammation-induced neurotoxicity in the PD model [23]. A novel GLP-1/GIP dual agonist was also able to reduce inflammation and enhance the Glial Derived Neurotrophic Factor (GDNF) release in the MPTP mouse model of PD [24]. In addition, Niacin decreased the expression of proinflammatory cytokines IL-1*β* and IL-6 via its receptor GPR109A to block the translocation of p-NF-*κ*B to the nucleus [25]. In this study, we found that pretreatment with crocetin distinctly decreased the expression of inflammatory associated genes (p-p65 and Pro-/Cleaved-caspase-1) and inflammatory cytokines (IL-1*β*, IL-6, IL-10, TNF-*α*, iNOS, and COX-2) in MPTP-induced PD mice and BV2 cells stimulated with MPP+. Moreover, the results of Iba-1 fluorescent staining also demonstrated the roles of crocetin in the regulation of microglia. Therefore, both *in vivo* and *in vitro* experiments suggest that crocetin treatment significantly inhibits the neuroinflammation in the MPTP-induced Parkinsonian mouse model.

A lot of evidence indicates that mitochondrial dysfunction is a vital factor in PD pathophysiology [26]. Mitochondrial protein import dysfunctions contribute to complex I-induced mitochondrial dysfunction and neurodegeneration in PD [27]. TRAP1 loss of function impacted MMP and damaged mitochondrial function in PD [28]. Curcumin protects against mitochondrial dysfunction and apoptosis in PINK1-deficient and paraquat-exposed cells, a cell model of PD [29]. In our study, crocetin treatment reversed the effect of MPP+ on the intracellular ROS levels, MMP, ATP content, and calcium content in BV2 cells. Additionally, MPP+ stimulation obviously increased the levels of Cyto in the cytoplasm, while crocetin treatment inhibited the increase. These results suggest that crocetin protects mitochondrial functions against MPP+ induced damage. Previous studies showed that the opening of mPTP produces a loss of thioretinaco ozonides from mitochondria, which impairs the ATP biosynthesis and causes mitochondrial dysfunction [30]. Therefore, we supposed that crocetin protected against mitochondrial dysfunction by regulating the mPTP viability. The results of mitochondrial swelling showed that crocetin treatment improved the mitochondrial swelling caused by the opening of mPTP. More importantly, we investigated that crocetin treatment blocked the interaction of ANT and Cyp D in mitochondria, which is key for the opening of mPTP [31]. These results suggest that crocetin inhibits the opening of mPTP through blocking the interaction of ANT and Cyp D.

Together, we have demonstrated that crocetin exerts antineuroinflammatory and neuroprotective effects both *in vivo* and *in vitro* on MPTP-induced experimental PD symptoms. Moreover, crocetin treatment protects against mitochondrial dysfunction by regulating the mPTP viability in the interaction of ANT and Cyp D dependent manner, suggesting that crocetin has the potential for Parkinsonian therapy.

## Figures and Tables

**Table 1:** A list of primers sequences for RT-PCR analysis.

Gene	Forward	Reverse
m-IL-1*β*	TGGACCTTCCAGGATGAGGACA	GTTCATCTCGGAGCCTGTAGTG
m-IL-6	TACCACTTCACAAGTCGGAGGC	CTGCAAGTGCATCATCGTTGTTC
m-IL-10	CGGGAAGACAATAACTGCACCC	CGGTTAGCAGTATGTTGTCCAGC
m-TNF-*α*	GGTGCCTATGTCTCAGCCTCTT	GCCATAGAACTGATGAGAGGGAG
m-iNOS	GCAGAATGTGACCATCATGG	ACAACCTTGGTGTTGAAGGC
m-COX-2	CAGACAACATAAACTGCGCCTT	GATACACCTCTCCACCAATGACC
m-*β*-actin	GATGGCCACGGCTGCTTC	TGCCTCAGGGCAGCGGAA

## Data Availability

The data used to support the findings of this study are available from the corresponding author upon request.
